# Response Variability in Transcranial Direct Current Stimulation: Why Sex Matters

**DOI:** 10.3389/fpsyt.2020.00585

**Published:** 2020-06-19

**Authors:** Thorsten Rudroff, Craig D. Workman, Alexandra C. Fietsam, John Kamholz

**Affiliations:** ^1^Department of Health and Human Physiology, University of Iowa, Iowa City, IA, United States; ^2^Department of Neurology, Carver College of Medicine, University of Iowa Hospitals and Clinics, Iowa City, IA, United States

**Keywords:** transcranial direct current stimulation, sex differences, hormones, cranial bone, cortical excitability

## Introduction

The past 20 years have seen an increased interest in non-invasive brain stimulation, such as transcranial direct current stimulation (tDCS), transcranial alternating current stimulation (tACS), and transcranial random noise stimulation (tRNS) (1). In tRNS, an alternating current is applied to the scalp with a constantly changing frequency, while tACS oscillates a sinusoidal current at a chosen frequency to interact with the brain’s natural cortical oscillations. Since the discovery that continuous weak direct current stimulation induces lasting excitability changes in the human motor cortex (2, 3), tDCS has been applied in various studies involving the induction and modulation of cortical neuroplasticity. It is generally accepted that anodal tDCS enhances cortical excitability (3) and cathodal tDCS decreases cortical excitability (inhibition) (4). However, this is an overly simplistic assumption and the effects of tDCS are likely much more complex.

The past 20 years have also witnessed a surge of findings concerning the influences of sex on many aspects of brain function and behavior, including emotion, memory, vision, hearing, facial processing, pain perception, navigation, neurotransmitter levels, and stress hormone action. The introduction of human brain-imaging techniques, like positron emission tomography (PET) and functional magnetic resonance imaging (fMRI), have heightened awareness of sex differences and revealed sex-related influences on brain functions that were previously thought to be similar between the sexes (5). For example, in 2006 Kuo et al. (6) suggested that sex differences pose a potential source of variability in cortical plastic changes from tDCS and should be addressed in cortical neuroplasticity manipulation studies in humans. However, in the intervening 15 years, the majority of tDCS studies have ignored biological sex differences. This neglect may account for some of the high inter-subject variability bemoaned by many tDCS investigators and has certainly delayed progress in the field. This is unfortunate because research into sex influences is vital to fully understanding the underlying mechanisms of non-invasive brain stimulation, especially for tDCS which has shown great variability (7–13). Indeed, approximately 50% of participants do not respond to tDCS and some even show effects contrary to the expected excitability response (e.g., inhibition from anodal tDCS and excitation from cathodal tDCS), evidenced by amplitude changes in motor evoked potentials (MEPs) elicited by transcranial magnetic stimulation (TMS) (14–16). Interestingly, Adenazato et al. (17) recently showed sex-related differences in cognitive Theory of Mind (ToM) abilities in healthy aging. Specifically, their findings showed that a single session of anodal tDCS over the medial prefrontal cortex led to significant slowing of reaction times in a communicative intention processing task in older women but not in men.

The purpose of this opinion article is to emphasize the consideration of biological sex, in perspective of physiological and anatomical considerations, on tDCS outcomes and to provide reflections and directions for future studies in the field. The focus of this paper is on transcranial direct current stimulation (tDCS), although the discussed issues can be applied to other transcranial electrical stimulation methods.

## Hormonal Levels and Neurotransmitter Balances

Hormonal levels fluctuate considerably more in women than in men and some studies exclude women from their research to reduce noise in their results (18). Men lack the cyclic fluctuation of sex hormones; testosterone and its metabolites, therefore, modulate cortical excitability similarly on different days. There are two main phases in the menstrual cycle of women: the follicular phase characterized by rising levels of estrogen and low levels of progesterone; and the luteal phase, which begins with ovulation and is accompanied by moderate levels of estrogen and high levels of progesterone. During the first follicular phase of menstruation (days 1–7), progesterone and estradiol levels are low and cortical excitation and inhibition from tDCS are less responsive. In the second follicular phase (days 7–14), estradiol increases while progesterone remains low, and excitability is enhanced and inhibition is reduced. In the first (days 14–21) and second luteal phases (days 21–28), when estradiol levels are moderate and progesterone levels are high, excitation is reduced and inhibition is enhanced (19–21). Thus, it appears, that progesterone drives the increase of cortical inhibition and estradiol enhances excitability. Additionally, measures of neurotransmitter levels important in tDCS responses (e.g., Gamma aminobutyric acid [GABA] and glutamate) support hormonal influences on cortical excitability. Using magnetic resonance spectroscopy (MRS), Epperson et al. (22) showed, in healthy women, that GABA concentrations in the primary visual cortex were lower in the luteal than in the follicular phase and that GABA was inversely correlated with both progesterone and estradiol levels. Similarly, another study found that glutamate concentrations in the medial frontal cortex were lower during the luteal than the follicular phase (23). Therefore, it is possible that assessing of the ratio of GABA/glutamate and estrogen/progesterone in future studies will confirm previous TMS findings that inhibition is increased during the luteal phase and reduced during the follicular phase, and excitation is reduced in the luteal phase and increased in the second follicular phase. Future tDCS studies that include women should carefully consider menstrual phases in their research designs, and women enrolled in repeated-session studies should be stimulated during the same phase of their menstrual cycle.

Supposing that an increase in cortical excitability is useful to enhance cognitive and motor function, negative consequences and non-optimal performances should be also noted. Overexcitation of the cortex, for example in the second follicular phase (i.e., excessive release of glutamate) might result in excitotoxicity and cell death (24, 25). However, it is noted that there have been no instances of seizures induced by tDCS using intensities ≤4 mA, which is considered safe for both men and women. Overexcitation likely depends on the targeted cortical region because the optimal excitation/inhibition (E/I) balance differs between brain areas. One location might exhibit overexcitation for a given set of tDCS parameters (e.g., intensity, duration, etc.) while others with different E/I levels, or an individual with lower excitation, may experience benefits (26). Indeed, the greater fatigability from 4 mA tDCS found in young women compared to young men by Workman et al. (27) could have been the result of overexcitation of the women. Excessive GABAergic inhibition also reduces neuronal output (28) and enhanced inhibition is correlated with higher network stability and reduced cortical plasticity (29).

A study by Stagg et al. (30) showed a significant correlation between the degree to which tDCS decreases GABA and a subject’s ability to learn a novel task. Specifically, those who demonstrated a greater decrease in GABA from tDCS also showed greater enhancements in learning. Therefore, a balance in the E/I interaction might be necessary to improve the efficacy of information transfer in the brain (31, 32). For tDCS, this indicates a specific dose-response association that interrelates with pre-existing baseline levels, which are unknown to the investigator. In addition to other confounding factors, this interaction could clarify the observed differences in tDCS outcomes and the great variability common in tDCS studies.

## Cortical Bone Structure

Another factor to consider is variations in skull structure/composition between men and women. This is important because a study by Vöröslakos et al. (33) found that only a small portion of the transcranial current reached the brains of human cadavers. They estimated that the soft tissues (e.g., scalp, subcutaneous tissue, and muscle) shunted ~50% of the current intensity and the skull shunted another 10–25%. Additionally, Russell et al. (34) modeled current intensities (from 0.5, 1, to 2 mA tDCS) arriving at the cortex of men and women using different sex-specific skull characteristics derived from real MR images. Their results revealed interesting sex-specific variations, with their model suggesting that men would receive ~45% more current at the cortex than women. The authors ascribed their results to the skull structure differences noted in the MRIs of men and women. Specifically, men had thicker skulls than women and the bone composition of the men was predominantly cancellous, while cortical bone was chiefly found in the women. Considering that cortical bone has a higher density than cancellous bone, the authors concluded that the denser cortical bone prevented more current from arriving at the cortex in the women. Consequently, possible differences in cortical bone density and cancellous bone thickness between men and women, and within each sex, should be considered in future tDCS study designs.

## Challenges, Solutions, and Future Directions

As noted above, some studies exclude women to control for the hormonal fluctuations and their effect on cortical excitation. Although this represents one potential solution, if the goal is to have an impact on the brain and behavior of the general population with tDCS, the correlation between the menstrual cycle and E/I must be explored further and women need to be included as participants. Hormonal levels measured from blood samples in women may provide indications of relative cortical excitability levels. However, more evidence is needed to validate the correlation between E/I and hormonal interactions. Another possible solution is to explicitly target the individual regional E/I balance by measuring GABA and glutamate levels, e.g., using MRS, and in so doing define the current polarity/parameters to optimize E/I. Although this method could be technically and practically challenging, such a study could carefully associate GABA and glutamate levels with other, more-easily measured individual characteristics (e.g., sex, age, hormone levels, etc.) to provide potential biomarkers of tDCS responsiveness.

For future research, it is particularly important that scientists plan for potential sex-related differences and that they choose their desired research populations with care to avoid unwanted noise in the data and/or, in extreme cases, the potential increased risk of seizure (35). This could be accomplished by accounting for these variables by pre-assessing E/I levels or activation patterns using neuroimaging methods (PET, fMRI, MRS), TMS, or at least behavioral patterns of performance. MRI or Computed Tomography (CT) could be also used to estimate cranial bone thickness and density and hormonal levels, especially in women, should be considered to avoid unwanted effects on tDCS cortical excitability. Specifically, systematic investigations to discover typical skull characteristics of men and women, and their association with cortical excitability, would help customize tDCS intensity by sex. tDCS should also be applied during specific phases of the menstrual cycle to optimize the effects of tDCS outcomes. For example, if tDCS is applied during the second follicular phase of the menstrual cycle, the intensity may need to be lower than in men to avoid overexcitation and create a positive outcome. Further individualization could then follow by measuring hormonal levels and bone composition of each individual and stimulating accordingly. This further strengthens the argument that tDCS should be customized for individual participants and discovering sex-related stimulation parameters would be a logical first step. It is noted that these sex differences are founded in *biological* variants inherent in men and women. Thus, future investigations should determine the *biological* sex of their subjects to control for the potential sources of variation discussed here.

The elucidation of ideal tDCS methodologies specific to each sex (e.g., low intensities for men, high intensities for women, or *vice versa*) constitutes a vital foundation for individualized tDCS application for participants. However, sex-related differences are only one of many issues (e.g. age, handedness, cognitive ability, neurological and psychiatric disorders, medications, recreational drugs, prior exposure to brain stimulation, electrode configurations, stimulation parameters, task dependency) suspected to contribute to the high variability in tDCS outcomes. Thus, it is questionable whether standardized tDCS applications are feasible, at least in the near future. Furthermore, it is virtually impossible to include all characteristics of every individual into clinical trial study designs to obtain homogenous samples, and tDCS tailored to individual participants is a more likely solution to response heterogeneity.

## Author Contributions

TR, CW, AF, and JK contributed to drafting the article and revising it critically for important intellectual content. All authors contributed to the article and approved the submitted version.

## Conflict of Interest

The authors declare that the research was conducted in the absence of any commercial or financial relationships that could be construed as a potential conflict of interest.
